# Epstein–Barr Virus-Induced Acute Pancreatitis—A Case Report and a Review of Literature

**DOI:** 10.3390/reports9030308

**Published:** 2026-09-11

**Authors:** Fiza Waseem, Aun Bin Tariq, Nahian Kazi, Muaad Reyad Abdulla, Ahmed H. Abdelhafiz

**Affiliations:** 1 Department of General Medicine, Rotherham General Hospital, Moorgate Road, Rotherham S60 2UD, UKaun.bintariq@nhs.net (A.B.T.);; 2 Department of Gastroenterology Medicine, Rotherham General Hospital, Moorgate Road, Rotherham S60 2UD, UK

**Keywords:** Epstein–Barr virus, pancreatitis, clinical presentation, diagnosis, management

## Abstract

**Background and Clinical Significance**: Epstein–Barr virus (EBV) is a common infection that rarely causes acute pancreatitis. EBV-associated acute pancreatitis is an uncommon but potentially serious complication that may result from direct viral invasion of pancreatic tissue or immune-mediated inflammation. Because its presentation can overlap with other causes of acute pancreatitis, EBV may be overlooked when common aetiologies are not identified. Recognising EBV as a potential cause is important for accurate diagnosis and appropriate management, particularly in patients presenting with pancreatitis alongside features of acute EBV infection; **Case Presentation**: We report a case of 58-year-old female with EBV-induced acute pancreatitis. She had a flu like illness two weeks prior to hospital admission, tested positive for EBV IgM antibodies and received supportive treatment by her general practitioner. However, her condition deteriorated, leading to hospital admission. She developed epigastric pain and investigations showed elevated serum lipase, impaired liver function tests and acute kidney injury. CT abdomen showed hepatosplenomegaly, lymphadenopathy and peripancreatic oedema consistent with acute pancreatitis. She was initially treated with antibiotics, however; her condition worsened and she developed severe hypotension requiring intensive care admission. Aggressive fluid resuscitation, inotropic support and continuous veno-venous haemofiltration (CVVH) were required. She gradually improved and subsequently was stepped down to the medical ward for supportive care and rehabilitation. She made full recovery and was discharged home after a 6-week length of stay; **Conclusions**: EBV-associated acute pancreatitis is an uncommon but potentially severe complication of EBV infection. EBV should be considered in patients with acute pancreatitis when common aetiologies have been excluded, particularly when pancreatitis occurs alongside features of acute viral infection. Early recognition and appropriate supportive management may be important in preventing or limiting severe complications.

## 1. Introduction and Clinical Significance

Epstein–Barr virus (EBV) is a double stranded DNA herpes virus. The virus has glycoproteins on its outer protein coat, which facilitate its entry into host cells. Virus transmission is mainly through the oral route through close personal contact and mainly affects children but also adults with slight female dominance [1]. After primary infection, the virus enters the epithelial cells and B-lymphocytes and can remain latent for life with intermittent shedding into circulation, especially in immunocompromised individuals. EBV infection is common. For example, a large-scale study in Taiwan showed an overall seropositive rate of 88.5%. The rate was 52.8% in children aged 2 years, 88.7% in those aged 5–7 years and 93.0% in those aged 14–16 years [2].

The prevalence is higher in overpopulated, low-income countries than in the western world [3]. The typical EBV clinical manifestations are wide, ranging from no symptoms to flu-like symptoms such as headache, fever, pharyngitis and malaise or specific manifestations such as splenomegaly and lymphadenopathy [4]. Occasionally, the disease may be associated with malignancies or present with severe systemic complications involving the respiratory, cardiovascular, genitourinary, gastrointestinal and nervous systems [5]. In addition, EBV can be associated with musculoskeletal manifestations such as rheumatoid arthritis, systemic lupus erythematosus, Sjögren syndrome and serum sickness-like reactions [6,7]. Common gastrointestinal manifestations of EBV infection are usually mild, such as transient impairment of liver function tests. However, acute pancreatitis is one of the uncommon EBV-induced severe gastrointestinal complications. The virus can directly invade the pancreatic cells or cause damage through induction of an immune reaction [8]. Although the known common causes of acute pancreatitis are alcohol or gall stone related, infectious causes can occur in about 10% of cases [9]. We present a case of EBV-induced acute pancreatitis, review similar published cases and highlight the importance of including viral cause in the differential diagnosis of acute pancreatitis.

## 2. Case Presentation

A 58-year-old female presented to the emergency department with complaints of fatigue, feeling hot, generalised weakness, increased urinary frequency and epigastric pain. She had a past medical history of obesity (body mass index 62 kg/m^2^), obstructive sleep apnea, fatty liver, depression, hypertension and osteoarthritis. Her medications included amlodipine, candesartan and citalopram. She was not on any immunosuppressive therapy. She denied excess alcohol intake, recent travel, any history of injuries or the use of over the counter or herbal medications. Ten days prior to her hospital presentation, she visited her general practitioner (GP) for flu-like symptoms. The GP treated her conservatively and sent for viral screening, which showed negative serology for hepatitis A, B, C and E. However, she was positive for EBV VCA IgM antibodies suggesting acute EBV infection. Her condition continued to deteriorate and lead to emergency department visit. General examination in hospital was normal with no pallor or jaundice. Cardiopulmonary examination was unremarkable. Abdominal examination revealed hepatomegaly with mild right and left upper quadrants tenderness. She was apyrexial with normal clinical observations.

**Figure 1:**
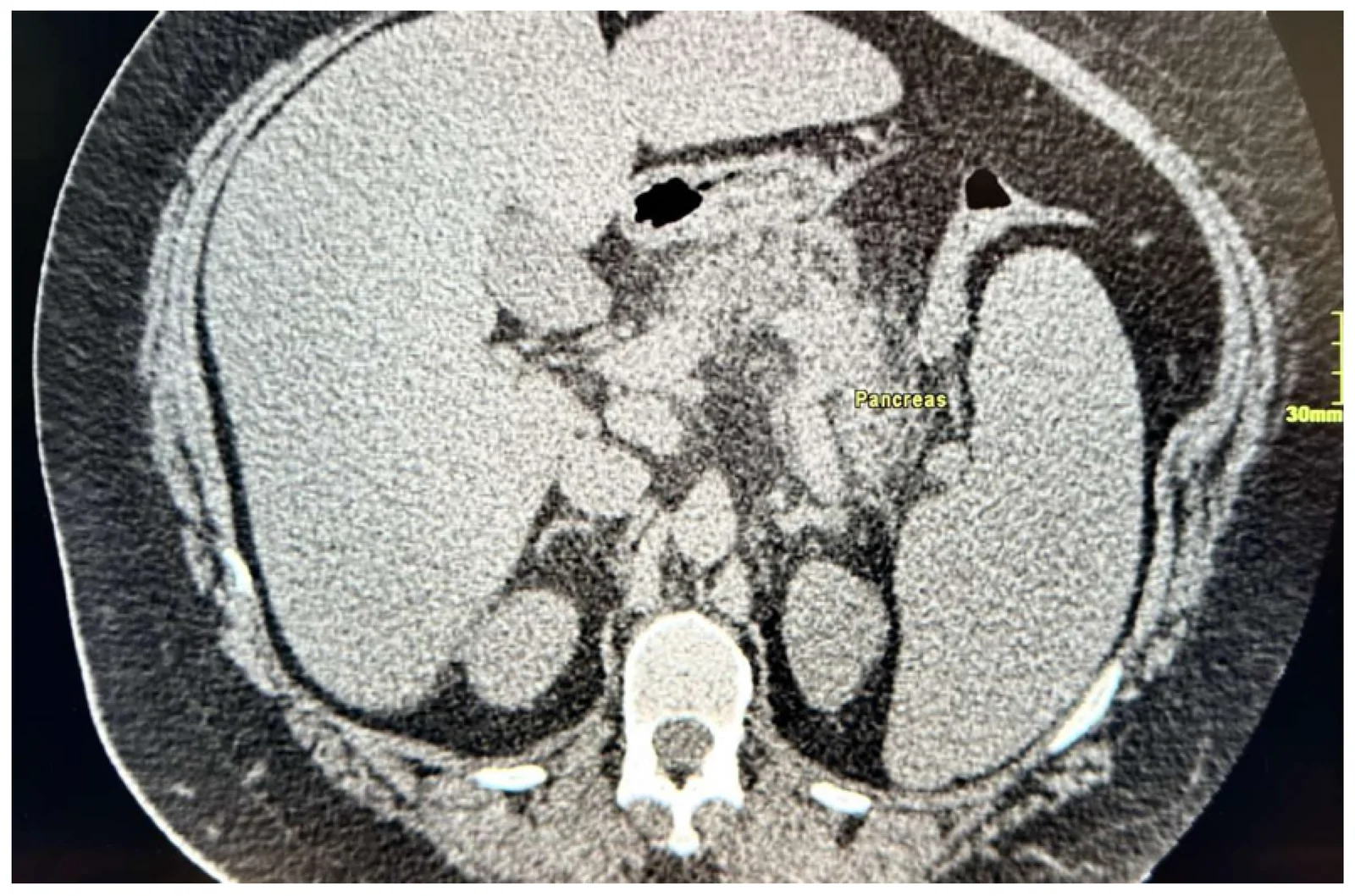
CT abdomen showing hepatosplenomegaly, lymphadenopathy and peripancreatic oedema and free fluid consistent with acute pancreatitis.

Investigations showed acute kidney injury (AKI) stage 3 with elevated serum creatinine (619 um/L) and urea (50.3 mmol/L). Liver function tests revealed elevated ALT (53 U/L), ALP (397 U/L), slightly raised bilirubin (21 umol/L) and CRP (141 mg/L). The lipidogram was within normal range. Serum lipase was high (682 U/L) consistent with acute pancreatitis. Full blood count showed normocytic anaemia (Hb 118 g/L, MCV 88.7 fL) normal total white cell count (4.8 × 10^9^/L), normal neutrophils (4.1 × 10^9^/L), low lymphocytes (0.7 × 10^9^/L) and low platelets (150 × 10^9^/L) Table 1 summarises laboratory results during her hospital stay. Virology was positive for EBV IgM antibody with EBV DNA detected on PCR using the Elitech system. The EBV PCR in this case was performed using the whole EDTA blood as the specimen. Regarding the primers, the assay used was the EBV ELITe MGB Kit. The assay targets a region of Epstein–Barr virus nuclear antigen 1 (EBNA-1) gene for EBV detection. The assay also includes an internal control targeting the promoter and 5′ untranslated region (UTR) of the human beta-globin gene to monitor amplification performance. Serological testing for HIV, hepatitis A, B, C, E and CMV antibodies were negative. Vasculitic and autoimmune screen panels were negative. Chest X-ray showed left basal atelectasis but lungs were clear. Abdominal ultrasonography revealed echo bright liver consistent with fatty infiltration but no evidence of cirrhosis or gallstones, splenomegaly and enlarged kidneys. CT urogram demonstrated hepatosplenomegaly, peripancreatic oedema and free fluid compatible with acute pancreatitis, as well as extensive retroperitoneal, iliac and inguinal lymphadenopathy. (Figure 1) The clinical impression was EBV-induced acute pancreatitis. Other diagnoses such as autoimmune pancreatitis or viral hepatitis were unlikely considering the negative screening.

She was initially treated with broad spectrum IV antibiotics to cover for possible sepsis but blood culture came back negative. However, she deteriorated later and developed hypotension (71/50 mmHg), persistently worse AKI (creatinine 626 um/L) and oliguria, prompting transfer to the critical care unit. She had fluid resuscitation, noradrenaline support and continuous veno-venous haemofiltration (CVVH) for 5 days. Her blood results showed hypocalcaemia requiring IV calcium replacement and nasogastric (NG) nutritional feeding was initiated. The patient was eventually successfully weaned off vasopressors and CVVH after 5 days. Her renal function gradually normalised and urine output improved. She subsequently stepped down to the medical ward for supportive care and rehabilitation. A multidisciplinary team, including haematology, gastroenterology and nephrology was involved in her care. EBV-induced pancreatitis was the most likely unifying diagnosis, and further invasive investigations such as lymph node or pancreatic biopsy, were deferred in favour of supportive management. She was discharged home after a total of 6 weeks of hospital admission.

## 3. Discussion

**Figure 2:**
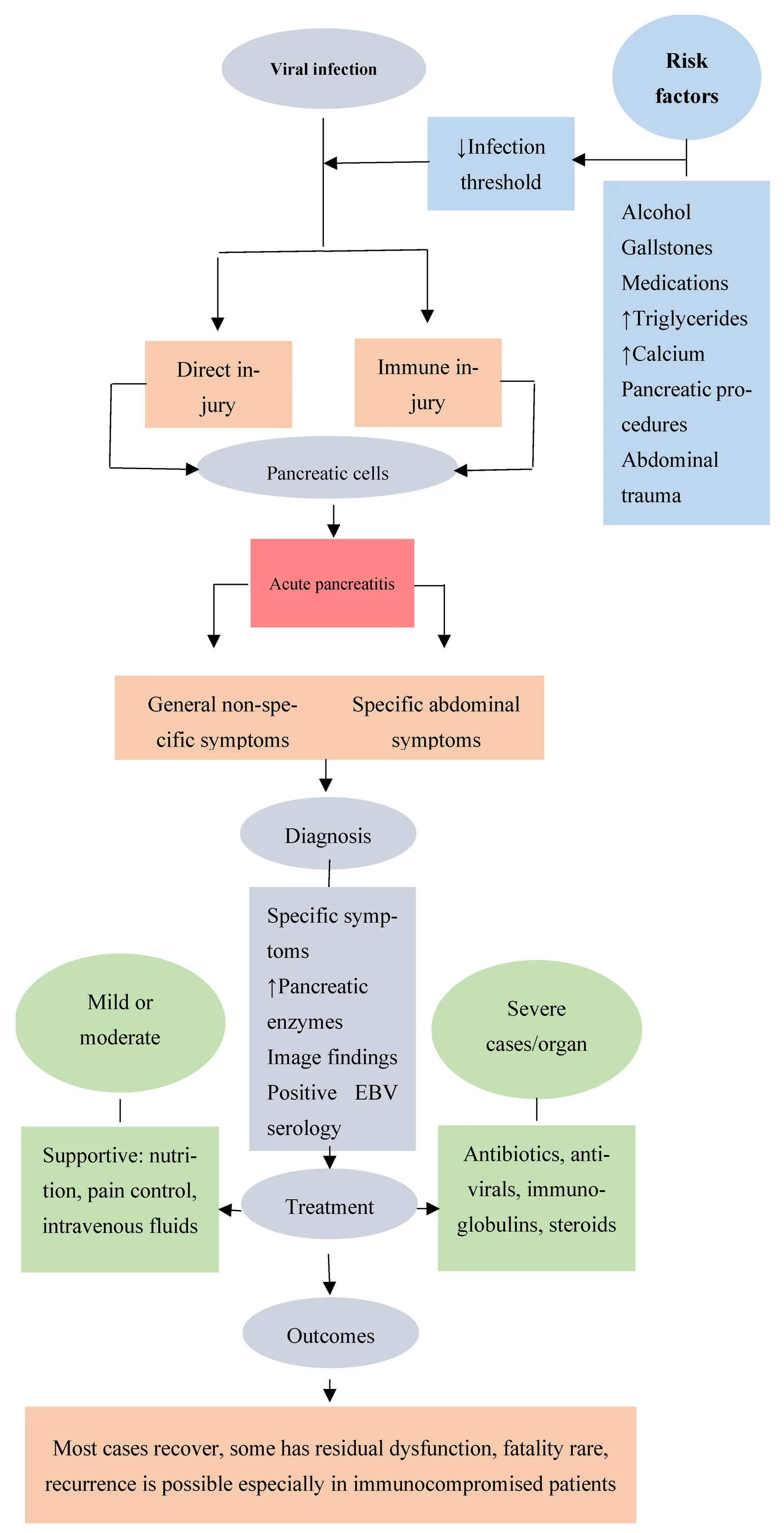
EBV-induced acute pancreatitis: mechanisms and clinical course. EBV = Epstein–Barr virus.

We have reported a case of EBV-induced acute pancreatitis in a 58-year-old woman. The aetiology of acute pancreatitis can be readily established in most patients and the most common causes include gallstones (40–70%) and alcohol (25–35%) [10]. However, infectious or virus-induced aetiology is uncommon. Among the infectious causes of acute pancreatitis, a systematic review reported that viruses were the leading aetiology (65.3%), then helminths (19.1%), bacteria (12.5%) and other organisms such as protozoa, mycobacteria and fungi accounted for 3.1% of cases [11]. Most common viral causes of acute pancreatitis include mumps virus, EBV, hepatitis virus, Coxsackie virus, echoviruses, cytomegalovirus, epidemic haemorrhagic fever virus, measles virus and human immunodeficiency virus [12]. However, EBV-induced acute pancreatitis is extremely rare, accounting for only 1.9% of cases [12]. Our diagnostic findings of EBV-induced acute pancreatitis are in accordance with the American College of Gastroenterology criteria including two of the following: abdominal pain, raised serum amylase and/or lipase greater than three times the upper limit of normal and positive abdominal imaging [10]. The definite gold standard diagnosis of EBV-induced acute pancreatitis requires pancreatic biopsy and testing for EBV-RNA in the pancreatic tissue [13]. However, diagnostic criteria of acute pancreatitis combined with evidence of recent EBV infection, in the absence of other causes of acute pancreatitis, is a reasonable and a pragmatic approach. The pathophysiology of EBV-induced and other viruses-related acute pancreatitis involves the viral entry into the pancreatic acinar cells through the action of angiotensin-converting enzyme 2 (ACE2). When the virus successfully enters the acinar cells, it forces the zymogen granules (containing digestive enzymes) to remain in the cell by preventing exocytosis. The zymogen granules combine with intracellular lysosomes to form autophagic vacuoles, which exert digestive damage to the pancreas [14]. Another proposed mechanism is indirect immune-mediated pancreatic inflammatory reaction to the EBV [8] (Figure 2).

EBV-induced pancreatitis typically occurs in young adults and up to 98.4% of cases occur under the age of 30 years [2]. EBV cases involve both young and adult patients with varying clinical presentations, diagnostic approaches and outcomes. We have searched the literature for case reports and defined young patients as ≤18 years (Table 2) and adults as >18 years old (Table 3). We searched across Medline, EMBASE and PubMed for case reports using search terms such as EBV, Epstein–Barr virus, acute pancreatitis, case reports, case studies and infection, individually and in combination. Th search was limited to English language publication and the search dates were from inception to present.

**Figure 3:**
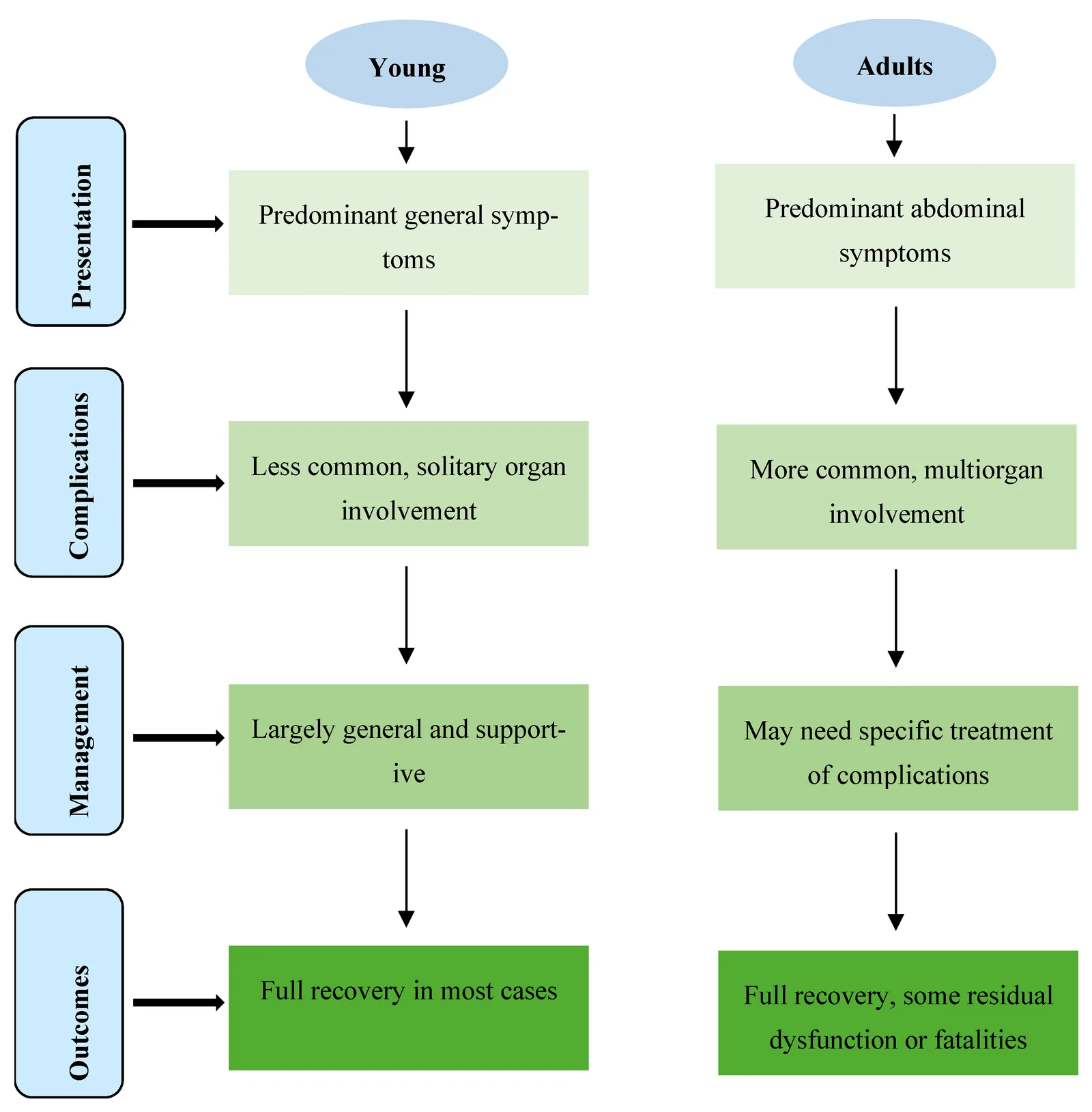
EBV-induced acute pancreatitis across young and adults.

In paediatric patients, EBV pancreatitis can occur in the absence of the typical clinical and haematological features of infectious mononucleosis. We found 11 cases of paediatric patients reported in the literature [5,15,16,17,18,19,20,21,22,23,24]. The age ranged from 3 to 18 years and seven (63.6%) cases were females. The main clinical presentations were abdominal pain, nausea and vomiting. The diagnosis was based mainly on serological and imaging findings. One case was diagnosed only on a clinical basis and raised pancreatic enzymes [21]. All cases were treated conservatively with nutritional support and symptom control. However, one case required the use of a combination of antibiotics and antiviral agents. The clinical course of this case was complicated by bilateral pneumonia, portal vein thrombosis and septic shock [16]. All cases recovered fully; however, seven (63.6%) developed complications. Among these cases, the most common complication was hepatobiliary involvement such as portal vein thrombosis, hepatocellular hepatitis, cholestatic hepatitis and cholecystitis. Two out of the seven cases were complicated by systemic inflammatory response syndrome leading to circulatory shock (Table 2). In adults, EBV pancreatitis may present with infectious mononucleosis symptoms, although infrequent, but pancreatitis features tend to predominate. Similar to paediatric patients, most cases recovered fully but mortality rate was high. Table 3 summarises eight adult cases reported in the literature [25,26,27,28,29,30,31]. The age ranged from 21 to 45 years and four (50%) cases were females. The primary clinical presentation was upper abdominal symptoms. All cases were diagnosed based on elevated pancreatic enzymes and positive EBV serology. Imaging was performed in six out of eight cases. Conservative management was sufficient in four cases; however, two cases required a combination of antiviral and antibiotics for treatment of pneumonia and hepatitis. One case required prednisolone for autoimmune haemolytic anaemia and the last case died quickly due to pancreatic necrosis and multiple organ failure and was treated conservatively. The complication rate was five out of eight cases (62.5%) and mortality was one out of eight (12.5%) (Figure 3).

It appears that EBV most commonly causes infectious mononucleosis type of illness and in rare occasions is complicated by acute pancreatitis. In general, several risk factors can predispose to acute pancreatitis, such as alcohol consumption, high triglycerides, hypercalcaemia, recent pancreatic procedures, abdominal trauma and medications. The presence of these factors at clinically insignificant levels can interact synergistically to lower the threshold for pancreatitis and potentiate EBV pancreatic penetration. This has been termed the “threshold model” where the risk factors interact, reaching a critical load to facilitate pancreatic infection [32] (Figure 2). EBV can involve other organs in addition to the pancreas and lead to complications. In children, hepatitis, cholecystitis, pneumonia, proctitis, portal vein thrombosis and septic shock, and in adults, gastritis, hepatitis, pneumonia, pleural effusion, pericardial effusion, ascites, autoimmune haemolytic anaemia and multi-organ failure have been reported [16]. Most cases of EBV acute pancreatitis recover with supportive treatment with no need for specific antiviral agents. However, EBV multiorgan infection may require specific antiviral treatment. In addition to antiviral agents, the use of steroids and immunoglobulins has been reported to achieve good outcomes in cases of EBV complicated by pneumonia, hepatitis and cholestasis [32]. Management of risk factors for pancreatitis should be part of the preventative strategies. However, recurrence of EBV acute pancreatitis is uncommon, but immunocompromised patients remain at risk [33]. The strength of this manuscript is the comprehensive review of the published cases of EBV-induced acute pancreatitis. However, it is limited by the fact that some of the cases included were diagnosed on clinical grounds and none of them had a confirmatory pancreatic biopsy due to the invasive nature of the procedure. In addition, there is no long-term follow-up of the cases included. There is also a limited literature on EBV reactivation and recurrence of acute pancreatitis but it remains uncommon and primarily associated with immune system compromise [33].

## 4. Conclusions

We presented a case of uncommon EBV-induced acute pancreatitis in adults. The patient was initially treated in the community for viral infection with no follow up plans. Clinicians should have a high index of suspicion and be aware that unexplained abdominal pain in patients with EBV infection should raise the possibility of acute pancreatitis. EBV-induced pancreatitis should be considered in patients presenting with pancreatitis, where common causes have been excluded, especially if accompanied by concurrent hepatitis, mild splenomegaly or atypical lymphocytosis on the blood film. Early supportive care in the correct setting may improve the outcome.

Infectious mononucleosis is a common EBV infection, presenting with flu-like symptoms, which is self-limited in most cases. EBV, however, may lead to gastrointestinal manifestations including acute pancreatitis. Clinicians should screen for acute pancreatitis in EBV infected patients presenting with abdominal pain. Treatment is supportive in most cases but follow up is required for a possible relapse especially in immunocompromised patients.

## Figures and Tables

**Table 1:** Blood results during hospital admission.

Days	Hb (119–149 g/L)	WCC (3.7–10 × 10^9^)	Plate (150–450 × 10^9^)	Neutr (1.7–6.6 × 10^9^)	Lymph (1.0–3.0 × 10^9^)	CRP (<5 mg/L)	Urea 2.5–7.8 mmol/L	Creat 44–71 (µmol/L)	ALT (10–49 U/L)	ALP (30–130 U/L)	Bil (0–20 µmol/L)	Lipase (12–53 U/L)
D 1	132	2.7	147	1.38	1.03	79	6.7	133	42	237	17	NA
D 10	118	4.8	150	4.1	0.7	141	50.3	619	53	397	21	682
D 15	111	5.7	89	4.2	1.0	140	42.6	458	129	364	22	NA
D 20	95	4.0	109	2.7	0.8	118	19.8	373	87	460	9	NA
D 30	104	6.8	197	5.5	0.8	2.0	6.2	70	49	210	8	NA

Hb = Haemoglobin, WCC = White cell count, Plate = Platelets, Neutr = Neutrophils, Lymph = Lymphocytes, CRP = C-reactive protein, Creat = Creatinine, ALT = Alanine transaminase, ALP = Alkaline phosphatase, Bil = Bilirubin, D = day.

**Table 2:** EBV-induced acute pancreatitis case reports in children.

Author, Year	Age (Years), Gender	Presentation	EBV Test	Pancreatic Enzymes	Imaging	Treatment	Complications, Outcome
Galzerano et al., 2014 [15]	3 F	Abdominal pain	VCA IgM+	A-3880 U/L L-NA	CT: Enlarged pancreatic head Wirsung’s duct ectasia	Fasting, parenteral nutrition, meropenem, teicoplanin, ganciclovir	Pneumonia, empyema, portal vein thrombosis, septic shock, recovered
Accomando et al., 2022 [16]	3 F	Abdominal pain, vomiting	VCA IgM+	A-913 U/L L-6450 U/L	US: Pancreatic enlargement	Fasting, parenteral nutrition, analgesics	None, recovered
Koutras et al., 1983 [17]	8 F	Abdominal pain, vomiting	VCA IgM+	A-300 U/L L-180 U/L	None	Symptomatic and supportive	Hepatitis, proctitis, recovered
Narchi et al., 2014 [18]	8 M	Abdominal pain, vomiting	VCA IgM+	A-80 U/L L-1000 U/L	MRI: Pancreas not visible	Fasting, intravenous fluids	Cholestatic hepatitis, cholecystitis, recovered
Kang et al., 2013 [19]	11 F	Abdominal pain, vomiting	VCA IgM+	A-4010 U/L L-4941 U/L	Oedema, peripancreatic fluid	Fasting, parenteral nutrition	Cholestatic hepatitis, recovered
Hedström et al., 1976 [20]	12 F	Abdominal pain	Clinical diagnosis	A-8700 U/L L-NA	None	Symptom control	None, recovered
López-Ibáñez et al., 2013 [21]	15 M	Abdominal pain	Heterophil antibody+	A-1251 U/L L-NA	CT: Globular pancreas, hepatosplenomegaly, ascites	NA	Hepatomegaly, splenomegaly, ascites, recovered
Werbitt et al., 1980 [22]	16 M	Abdominal pain, nausea, vomiting	VCA IgM+	A-378 U/L L-NA	Normal CT	Conservative	None, recovered
Khawcharoenporn et al., 2008 [23]	18 F	Abdominal pain	VCA IgM+	A-620 U/L L-659 U/L	Oedematous pancreas	Symptom control	Cholecystitis, hepatitis, DIC, septic shock, recovered
Hammami et al., 2019 [5]	18 F	Abdominal pain, nausea	VCA IgM+	A-327 U/L L-2016 U/L	CT: Signs of acute pancreatitis	Analgesia, hydration and a slowly advanced diet	Hepatitis, recovered
Wislocki et al., 1966 [24]	18 M	Abdominal pain, vomiting	Heterophil Ab+	A-480 U/L L-NA	None	Fasting, IV fluids, analgesics	None, recovered

EBV = Epstein–Barr virus; F = Female; + = Positive; VCA = Viral capsid antigen; A = Amylase; L = Lipase; CT = Computed tomography; NA = Not available; US = Ultrasound; M = Male; MRI = Magnetic resonance imaging; DIC = Disseminated intravascular coagulation; Ab = Antibody; IV = Intravenous.

**Table 3:** EBV-induced acute pancreatitis case reports in adults.

Author, Year	Age (Years), Gender	Presentation	EBV Test	Pancreatic Enzymes	Imaging	Treatment	Complications, Outcome
Singh et al., 2016 [25]	21 F	Abdominal pain, nausea, vomiting	VCA IgM+ Positive monospot test	A-NA L-4301 U/L	CT: pancreatic oedema	Supportive, prednisone	Autoimmune haemolytic anaemia, hepatitis, recovered
Jahann et al., 2012 [26]	22 M	Abdominal pain	VCA IgM+	A-330 U/L L-2300 U/L	NA	Symptom control	None, recovered
Hedström et al., 1976 [20]	24 M	Fever, abdominal pain, sore throat, nausea.	Positive monospot test, heterophile Ab+	A-376 U/L L-NA Urine A-4700 U/L	NA	Symptom control	None, recovered
Cook et al., 2015 [27]	25 M	Abdominal pain, nausea, fever	VCA IgM+	A-NA L-429 U/L	CT: pancreatic oedema	Supportive	Hepatitis, pleural effusion, ascites, recovered
Coffin et al., 2025 [28]	25 M	Recurrent abdominal pain	EBNA+, EBV VCA IgG+, EBV VCA IgM+	Elevated lipase	MRI: diffuse inflammation	Supportive	None, recovered
Zhu et al., 2017 [29]	35 F	Abdominal pain, vomiting	VCA IgM+, heterophile Ab+	A-1300 U/L L-1450 U/L	CT: pancreatic oedema	Fasting, GI decompression, somatostatin, parenteral nutrition, acyclovir, antibiotics.	Hepatitis, pneumonia, recovered
Fiani et al., 2021 [30]	35 F	Abdominal pain, fever	VCA IgM and IgG+, EBV DNA+	A-129 U/L L-408 U/L	CT: enlarged pancreas	Bowel rest, hydration, mechanichal ventilation, antibiotics, antivirals, steroids	Hepatitis, pneumonia, respiratory failure, recovered
Huang et al., 2021 [31]	45 F	Abdominal pain	EBV DNA+	Elevated A and L up to 3 times	CT: pancreatic necrosis	Supportive	MOF, pericardial and pleural effusions, died

EBV = Epstein–Barr virus; F = Female; + = Positive; VCA = Viral capsid antigen; A = Amylase; L = Lipase; CT = Computed tomography; M = Male; NA = Not available; EBNA = Epstein–Barr nuclear antigen; MRI = Magnetic resonance imaging; Ab = Antibody; GI = Gastrointestinal; MOF = Multi-organ failure.

## Data Availability

The original contributions presented in this study are included in the article. Further inquiries can be directed to the corresponding author.
